# A multi-center cross-sectional study to explore cancer patients’ views on facilitators and barriers for the implementation of oncological exercise therapy

**DOI:** 10.3389/fonc.2026.1800417

**Published:** 2026-06-10

**Authors:** Dominik Morlok, Laura Bieniosek, Hansjörg Baurecht, Melanie Reitz, Thomas Okon, Janina Meuer, Christine Welker, Wolfgang Herr, Tobias Pukrop, Sabine Einhell, Anika Berling-Ernst, Annalena Wehner, Mirko Brandes, Sebastian Theurich, Bernardine Madl, Louisa Sell, Thorsten Schmidt, Patrick Jahn, Carlotta Evers, Rebecca Zimmer, Renee Stark, Hajo Zeeb, Michael Leitzmann, Freerk T. Baumann, Anne Herrmann

**Affiliations:** 1 Department of Epidemiology and Preventive Medicine, Medical Sociology, University of Regensburg, Regensburg, Germany; 2 Department of Epidemiology and Preventive Medicine, University of Regensburg, University Hospital Regensburg, Regensburg, Germany; 3 Department I of Internal Medicine, Center of Integrated Oncology Aachen Bonn Cologne Düsseldorf, University Hospital of Cologne, Cologne, Germany; 4 Department of Prevention and Evaluation, Leibniz Institute for Prevention Research and Epidemiology – BIPS GmbH, Bremen, Germany; 5 Department of Internal Medicine III, University Hospital Regensburg, Regensburg, Germany; 6 Bavarian Center for Cancer Research, Bayerisches Zentrum für Krebsforschung (BZKF), Regensburg, Germany; 7 Center for Translational Oncology, University Hospital Regensburg, Regensburg, Germany; 8 Bavarian Center for Cancer Research (BZKF), Munich, Germany; 9 Department of Medicine III, Ludwig Maximilians University (LMU) University Hospital, Ludwig Maximilians University (LMU) Munich, Munich, Germany; 10 Comprehensive Cancer Center Munich, Ludwig Maximilians University (LMU) University Munich, Technical University of Munich, Munich, Germany; 11 Technical University of Munich, School of Medicine and Health, Department for Preventive Sports Medicine and Sports Cardiology, Technical University of Munich (TUM) University Hospital, Munich, Germany; 12 University Cancer Center Schleswig-Holstein, Kiel, Germany; 13 Health Service Research Working Group, Acute Care, Department of Internal Medicine, University Medicine Halle (Saale), Halle (Saale), Germany; 14 University Cancer Center Hamburg, University Medical Center Hamburg-Eppendorf, Hamburg, Germany; 15 Pediatrics III, Department of Pediatric Hematology/Oncology, West German Cancer Center, University Hospital Essen, Essen, Germany; 16 Professorship of Public Health and Prevention, School of Medicine and Health, Technical University of Munich, Munich, Germany

**Keywords:** oncological exercise therapy, physical activity, cancer, barriers, implementation, facilitators

## Abstract

**Background:**

Oncological exercise therapy (OET) can help improve various patient outcomes, including fatigue and quality of life, and in certain cancer types, progression-free and overall survival. Despite this, OET is not routinely implemented into clinical practice. A better understanding of cancer patients’ perceived barriers and facilitators to participating in OET may support its integration into routine care. This multi-center cross-sectional study investigating cancer patients’ views will help close this evidence-practice-gap.

**Methods:**

As part of IMPLEMENT, a nationwide OET implementation research project in Germany, an exploratory study with a quasi-experimental design was conducted using a non-representative sample of consenting cancer patients. Participants completed a survey either online or in paper format. A comprehensive descriptive analysis was conducted using SPSS and RStudio.

**Results:**

A total of 402 cancer patients provided a questionnaire (61% female; mean age 57 years, SD 15). The most common cancer types were breast cancer (34%), lymphoma or leukemia (12%), and colon cancer (5%). Most patients reported feeling physically (79% agreed or somewhat agreed) and mentally (85% agreed or somewhat agreed) capable of participating in OET. Motivation to engage in OET was perceived to be high (76% agreed or somewhat agreed), and patients reported having sufficient time to become physically active (83% agreed or somewhat agreed). However, only half of participants said they were offered OET by their healthcare providers (48% agreed or somewhat agreed).

**Conclusion:**

Our findings indicate that most cancer patients are both willing and able to participate in OET. However, they are not routinely informed about OET. Therefore, tailored strategies for improving patient access to OET are needed.

**Trial registration:**

Clin. Trials: NCT06496711. German Clinical Trial Register (Deutsches Register Klinischer Studien - DRKS): 00032292. Bavarian Cancer Research Center (Bayerisches Krebsforschungszentrum - BZKF resp. ZKS): DZ-2024-2165-9.

## Introduction

Globally, cancer is among the top 10 causes of death in upper-middle and high-income countries (1). In Germany, approximately 231,400 women and 261,800 men received a cancer diagnosis in 2020. Over 1.6 million people are currently living with and beyond cancer, with numbers expected to rise due to an ageing population (2). This trend demands further advances in therapy to enhance both cancer-specific treatment outcomes and quality of life.

In addition to standard medical treatment, oncological exercise therapy (OET), an evidence-based exercise program tailored to cancer patients, may enhance physiological parameters like fitness levels, body composition including muscular capacities and function, and cardiorespiratory outcomes (3–5). Also, psychosocial benefits were confirmed for physically active participants, including increases in quality of life, self-image, reduced cancer-related fatigue, and depressive symptoms compared to inactive control groups (3, 5–7). Both a meta-analysis and a RCT recorded life-prolonging effects of physical activity (PA): Risk of death decreased for patients with breast cancer (8) and disease-free survival was significantly higher in colon cancer patients (9) when assigned to a structured exercise program.

Despite substantial evidence for the beneficial effects of PA and OET, OET remains underused in routine oncology care in Germany, highlighting a significant evidence-practice-gap (10, 11). Although many health care providers are aware of the benefits of exercise therapy, detailed programs are rarely recommended and structural availability and cooperations with qualified facilities remain scarce (10). OET is used by less than half of all eligible cancer patients in Germany (12).

Due to limited evidence regarding the implementation of OET in Germany, eight university hospitals and research institutions initiated the German Cancer Aid-funded 3-year IMPLEMENT project to bridge this gap. They aim to promote OET as a routine treatment option in cancer care by developing implementation strategies to increase the number of patients participating in OET (11). This study is part of the IMPLEMENT project’s baseline survey and investigates patient-perceived barriers and facilitators to the successful implementation of OET into routine practice. Specifically, it examines German cancer patients’ perspectives and discusses potential strategies to improve patient access to OET.

## Methods

### Design and setting

For this multi-center cross-sectional study, a questionnaire was distributed in eight university hospitals and research institutions located in five different federal states across Germany (the “IMPLEMENT sites”, details at https://cio.uk-koeln.de/leben-mit-krebs/bewegung/studien-und-publikationen/implement-projekt/). The study was conducted from November 2023 to June 2024. A detailed description of the methodology is provided in the published study protocol (11).

### Participants

Eligible patients were adults (aged 18 years or older) with any cancer diagnosis who were undergoing medical treatment or receiving follow-up care. Patients were required to be fluent in German (as determined by research staff) and to attend an outpatient appointment at one of the participating hospitals. Patients were excluded if they were unable or unwilling to provide informed consent.

### Recruitment and data collection

Ethics approval for IMPLEMENT was initially obtained from the University of Bremen, Germany (ref. 2023-16). All participating institutions received ethics approval from their respective ethics committees based on this initial authorization. Study procedures adhered to principles of Good Clinical Practice (GCP) and the Declaration of Helsinki. The study is registered at the Clinical Trials registry (NCT06496711), at the German Clinical Trials registry (DRKS00032292) and at the Bavarian Cancer Research Center (BZKF or ZKS: DZ-2024-2165-9). At each university hospital, research assistants were responsible for patient recruitment and data collection. They were supported by network partners and relevant stakeholders at each IMPLEMENT site, including treating physicians and nurses. Sampling methods across sites did not differ systematically in recruitment intensity, although some university hospitals had higher patient volumes, which led to a greater number of completed questionnaires. Research assistants provided oral and written study information and handed out questionnaires. Eligible patients provided informed consent before completing the questionnaire either in pen-and-paper format or online via QR codes provided on flyers. Patient participation was voluntary and anonymous. Due to the study design and missing registry data, the total number of eligible patients could not be assessed.

### Measure development

A multidisciplinary team involving experts in oncology, physiotherapy, sports science, health services research, psychology, nutrition and communication science developed a questionnaire. Item selection was based on a review of the available literature on exercise behavior and barriers and facilitators to exercise therapy. To ensure face validity, the draft questionnaire was reviewed by healthcare professionals and researchers, leading to revisions of selected items to improve understandability. A sub-sample of 10 patients with cancer were recruited by research assistants of three different IMPLEMENT sites to pilot test the questionnaire for comprehensibility, completion time and relevance. Only minor adjustments were made to simplify questions and improve filtering.

### Measures

The questionnaire included items assessing patients’ socio-demographic characteristics, cancer diagnosis, treatments received, leisure-time PA (Godin-Shephard Leisure-Time Exercise Questionnaire – short form), knowledge about PA, motivation and intention to become physically active, as well as quality of life (EORTC QLQ-C30). In addition, patients were asked about current and previous OET experiences and whether they received information about OET, including from whom and whether they deemed this information to be sufficient. Further items were included to explore patients’ views of barriers and facilitators to attending OET. These items addressed potential barriers and facilitators at individual, structural, and informational levels. Patients responded on a four-point Likert scale (1= disagree, 2= somewhat disagree, 3= somewhat agree, 4= agree) in order to avoid moderacy response bias, with an additional option of “not applicable”.

### Analysis

Analyses were conducted using IBM SPSS 29 and R 4.4.1 (RStudio), applying a complete case approach. Frequencies and percentages were calculated for each item. Associations between participation in OET and sociodemographic factors (age, gender, diagnosis) as well as perspectives on barriers and facilitators were assessed using Chi-squared tests (χ^2^).

## Results

### Sample

A total of 402 cancer patients returned a questionnaire. This corresponds to a consent rate of 13% based on all distributed paper questionnaires and QR-code invitations. Percentages are calculated based on the total study population (n=402). Thirty-six records (9%) had missing items regarding barriers and facilitators. In subgroup analyses, percentages are calculated based on the respective subgroup population. The mean age was 57 years (standard deviation SD ± 17), with 61% identifying as female (n=244). The median net income category was 3000€-4000€ per household. 62% had a high school diploma or university entrance qualification (n=248), 26% had an intermediate school leaving certificate (n=103) and 10% left school with a lower secondary school certificate (n=38). Most patients were diagnosed with breast cancer (34%, n=136), lymphoma or leukemia (12%, n=47), or colon cancer (5%, n=21) and 69% were or had been receiving treatment for cancer (n=279). Of those, 44% were receiving treatment at the time of data collection (n=175/278) and 26% received their last treatment more than half a year ago (n=103/278). **Table 1** provides a summary of patients’ socio-demographic, disease- and treatment-related characteristics, leisure-time PA and participation in OET.

**Table 1:** Participant sociodemographics and disease profile (n=402).

Participants n (%)	
Gender	
Female	244 (60.7%)
Male	153 (38.1%)
Diverse	1 (0.2%)
Missing	4 (1%)
Age in years	
<30	21 (5.2%)
30-39	31 (7.7%)
40-49	51 (12.7%)
50-59	104 (25.9%)
60+	191 (47.5%)
Missing	4 (1%)
Health insurance	
Public	325 (80.8%)
Private	73 (18.2%)
Missing	4 (1%)
Education	
Lower secondary school certificate	38 (9.5%)
Intermediate secondary school certificate	103 (25.6%)
Higher education entrance qualification/High school diploma (A-level equivalent)	248 (61.7%)
Other school qualification	3 (0.7%)
No school qualification	2 (0.5%)
Still a student	1 (0.3%)
Missing	7 (1.7%)
Migration background	
No	361 (89.8%)
Yes	29 (7.2%)
Missing	12 (3%)
BMI	
Underweight (<18.5)	7 (1.7%)
Normal weight (<25)	219 (54.5%)
Overweight (<30)	112 (27.9%)
Obesity (≥30)	44 (10.9%)
Missing	20 (5%)
Cancer type	
Breast	136 (33.8%)
Lymphoma/leukemia	47 (11.7%)
Colon	21 (5.2%)
Other	185 (46.0%)
Missing	13 (3.3%)
Participation in OET for cancer patients	
Yes	192 (47.8%)
No	192 (47.8%)
Missing	18 (4.4%)

BMI, body mass index; OET, oncological exercise therapy.

### Low level of individual barriers

**Figure 1:**
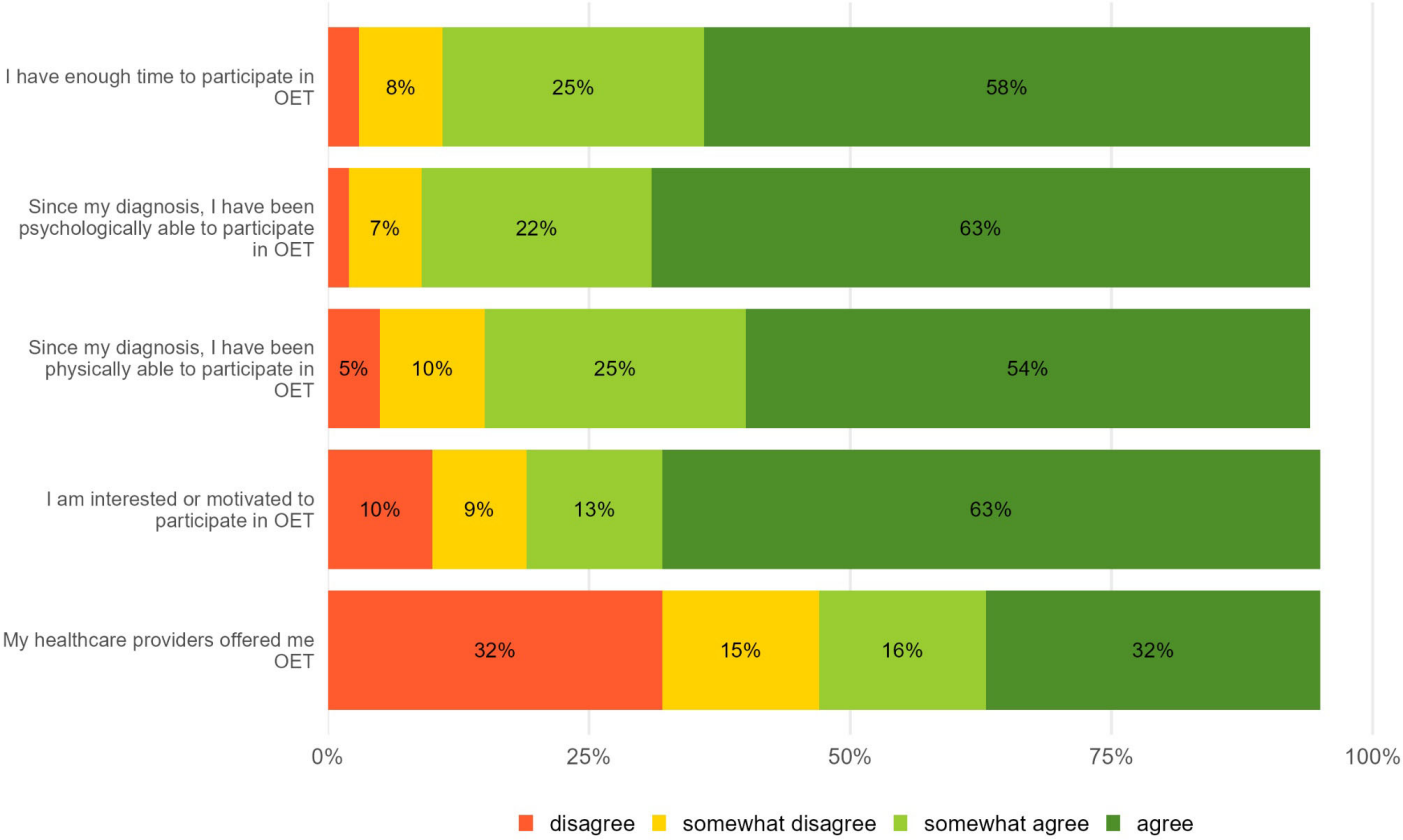
Stacked bar chart of key barriers and facilitators to participating in OET. Percentages are based on the total study population (n=402). Missing responses were 6% for time to participate, 6% for psychological ability to participate, 6% for physical ability to participate, 5% for motivation to participate, and 5% for OET being offered by healthcare providers.

Most cancer patients reported feeling physically capable of participating in OET (79% agreed or somewhat agreed, n=319). Even more patients indicated that they felt psychologically capable of participating in OET (85% agreed or somewhat agreed, n=343). Moreover, motivation to engage in OET was perceived to be high (76% agreed or somewhat agreed, n=306), and patients reported having sufficient time to become physically active (83% agreed or somewhat agreed, n=332) (**Figure 1**). Furthermore, a small proportion of patients (11% of OET participating patients, n=22/192) reported low motivation but nonetheless participated in OET. Nine patients who perceived themselves as physically unable to engage in OET nevertheless participated (5% of OET participating patients, n=9/192). Of those who took part in OET, 21% rated their overall health as poor (n=41/192) and 20% stated that their quality of life was poor (n=39/192). 54% of patients who reported metastases (n=73/133) participated in OET, while 45% of patients with metastases did not participate (n=60/133).

Most patients denied needing the support of their caregivers to participate in OET: Only 8% (n=30) of patients agreed or somewhat agreed to the statement “I only take part in sports and exercise therapy if my carers (e.g. spouse, friends) advise me to do so”.

Around half of patients proactively asked their healthcare providers for a consultation on PA and referral to OET (47% agreed or somewhat agreed, n=190).

### Relevance of structural and organizational barriers

Most patients indicated that costs did not hinder them from attending OET (70% agreed or somewhat agreed, n=283). More than half of the patients did not agree with the statement that “there is no time for the topic of OET in doctor-patient-communication” (56% disagreed or somewhat disagreed, n=225). 56% of patients reported that they felt their physician (agreed or somewhat agreed, n=223) and 54% agreed that other medical staff (agreed or somewhat agreed, n=215) has adequate knowledge of OET. Almost every fourth patient (22%) agreed or somewhat agreed (n=87) that public transport accessibility is lacking. Less patients regarded accessibility by car to not being sufficient (15% agreed or somewhat agreed, n=58). Yet 47% of patients agreed or somewhat agreed to the statement that there are too few OET programs that meet their needs (n=189).

### Limited information and limited referral to OET

Around two-thirds of patients have been informed about the benefits of exercise (66% agreed or somewhat agreed, n=266). However, only 54% (n=217) felt sufficiently informed about the topic. Moreover, only half of the participants stated that they had been offered OET by their healthcare providers (48% agreed or somewhat agreed, n=193).

Some patients had received positive reports about OET from other cancer patients who had previously participated (42% agreed or somewhat agreed, n=169).

### Reported key barriers of patients not participating in OET

**Figure 2:**
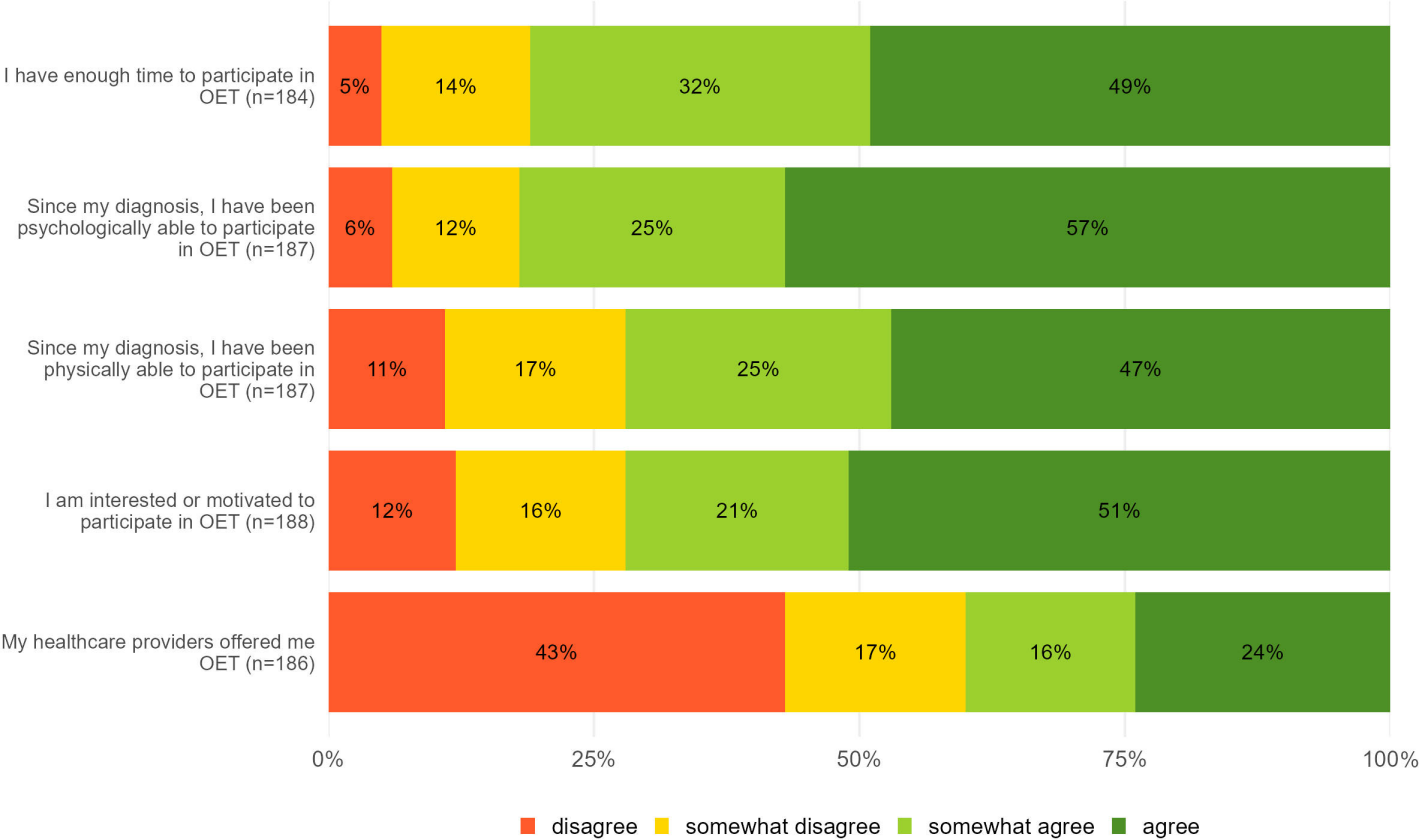
Stacked bar chart illustrating key barriers among patients not participating in OET.

If patients did not participate, they mentioned the following key reasons: 57% of patients agreed or somewhat agreed to the statement that there are too few OET programs that meet their needs (n=105/183). Around half of the patients who did not participate in OET perceived that the medical staff at their treatment facility had limited knowledge of OET (49% agreed or somewhat agreed, n=90/182). Most non-participants in OET have been informed about the benefits of exercise (61% agreed or somewhat agreed, n=114/187). However, only 47% (n=88/189) felt sufficiently informed about the topic. Although most patients reported having enough time to participate in OET (81% agreed or somewhat agreed, n=149/184), being motivated (72% agreed or somewhat agreed, n=135/188) and psychologically (82% agreed or somewhat agreed, n=154/187) as well as physically (72% agreed or somewhat agreed, n=135/187) able to participate in OET, less than half of the participants stated that they had been offered OET by their healthcare providers (40% agreed or somewhat agreed, n=174/186) (**Figure 2**). Compared to participants in OET, non-participants reported statistically significant higher barriers to exercising. They felt less physically able to participate and less informed by their healthcare providers, with differences of 23% (95% agreed or somewhat agreed compared to 72%) and 22% (62% agreed or somewhat agreed compared to 40%) respectively (**Table 2**).

**Table 2:** OET participation: patient comparison.

Variables (“agreed or somewhat agreed”)	OET participants (%)	Non-OET participants (%)	Percentage point difference
I have enough time to participate in OET	95 (n=192)	81 (n=184)	14
Since my diagnosis, I have been psychologically able to participate in OET	98 (n=192)	82 (n=187)	16
Since my diagnosis, I have been physically able to participate in OET	95 (n=192)	72 (n=187)	23
I am interested or motivated to participate in OET	88 (n=192)	72 (n=188)	16
My healthcare providers offered me OET	62 (n=192)	40 (n=186)	22

OET, oncological exercise therapy.

### Correlation analysis

Participation in OET was moderately to strongly associated with motivation (Cramer’s V = .244) and physical ability (V = .228), followed by psychological ability (V = .196), available time (V = .194), and whether OET was offered by a healthcare provider (V = .176). All associations were statistically significant (p<.001). There was no significant association between participation in OET and type of health insurance (V = .024, p=0.89).

To assess age-related influence, associations between age-categories and reported barriers and facilitators were examined using Chi-squared tests (χ^2^). No significant differences were found between age groups, with one exception: a moderate association emerged regarding the perceived physical ability to participate in OET (Cramer’s V = .176, p<.005). Patients in the older age groups were more likely to report feeling physically less capable of engaging in OET.

Also, when tested for gender, there was a weak correlation with women being more likely to receive information from other patients, having been given positive reports about OET from other cancer patients who have already taken part in OET (V = .150, p<.005).

There was a moderate association between being informed about the benefits of PA and cancer diagnosis (V = .297, p<.005) with 82% of breast cancer patients being informed, 67% of patients with lymphoma and 63% of others. Another moderate association was found between proactively asking for a consultation and referral to OET and cancer diagnosis (V = .293, p<.005) with 54% of breast cancer patients asking for a referral on their own initiative. Yet only 41% of patients with lymphoma and 45% of others asked for a referral by themselves.

## Discussion

### Summary of results

This study comprehensively investigated cancer patients’ perspectives on facilitators and barriers for the implementation of OET using a heterogeneous sample drawn from a multi-center cross-sectional study. Results suggest that most cancer patients are both willing and able to participate in OET and have sufficient time to do so. Furthermore, patients do not experience costs as a relevant barrier to becoming physically active. Nevertheless, patients are not routinely informed about the availability of OET. Patients who did not participate in OET reported statistically significantly higher barriers to becoming physically active than those who did participate in OET.

### Relevance of barriers and facilitators compared to other studies

Results from a systematic review by Clifford et al. indicate that the most prominent barriers to becoming physically active experienced by cancer patients are treatment-related side effects (e.g. fatigue) and lack of time. Motivational aspects were also perceived as a relevant barrier (13). This is in line with further reviews highlighting a lack of wellbeing and interest as common reasons for not joining OET in clinical settings (14, 15). Similar findings have been reported for German cancer patients (16). This stands in partial contrast to some of our study findings indicating that patients’ perceived individual barriers (e.g., motivational, time, fatigue) were of less relevant to become physically active than barriers related to information and communication.

Although around two-thirds (66%) of patients reported receiving information about the benefits of exercise, only 54% felt adequately informed on exercise. This is in line with other studies pointing to the crucial role of information and communication in helping people become physically active (17–19). Particularly, the importance of physicians’ exercise counseling in patients with cancer has been stressed by previous research in this area (20, 21). Moreover, patients typically engage with multiple healthcare providers. However, our study results indicate that only about half of patients received an actual offering of OET by their healthcare providers (48%). This finding aligns with previous reports, indicating that only one in two cancer patients were recommended exercise by a healthcare provider (16). Correlation analysis indicated a weak but existing association between OET offering by a health care provider and participation.

Highlighting the reasons patients reported for not participating in OET may enable a more focused anamnesis and tailored questioning, which could help to address individual needs more effectively. This may also improve adherence to prescribed care, including exercise therapy (22, 23).

Further knowledge of cancer patients’ barriers and facilitators to exercise could also provide guidance for refining and tailoring OET.

### Evidence-based strategies to help overcome deficits in communication

Our results highlight that further strategies are needed to help improve information and communication about exercise and OET. Individually tailored counselling by healthcare providers may be particularly effective in increasing the uptake of exercise (24). This may be improved through evidence-based training programs on increasing knowledge around the benefits and access to PA (20). Also, information should be individually tailored accounting for patients’ disease status and prior exercise experiences (20, 25). Exercise referral pathways to OET may be a time efficient strategy to address this (26). Social prescribing could be a promising approach to provide patients with OET offers that meet their individual needs and preferences. It may allow a more adequate evaluation of patients´ capabilities and resources, and help to meet their needs by employing non-medical and community-based support, e.g., transport to/from the clinic or access to local exercise offers. In cancer care, social prescribing links patients to non-clinical community resources to improve wellbeing and address psychosocial needs, primarily via a link worker (27).

Another approach to address patients’ needs is to provide information about OET and PA on digital platforms/applications. 47% of patients reported that there are too few OET programs that meet their needs. Easily accessible information online about diverse OET offers may not only reduce the informational burden on healthcare professionals but also enhance patients’ self-efficacy. The need for easy access to healthcare information for patients, their families, and other interested individuals through various media formats, including text, videos, and e-learning tools is growing (28). Digital platforms and mobile applications can have a relevant impact in promoting patient engagement and adherence to treatment programs by providing easily accessible resources including e.g. interactive Q&A tools to support information needs (29). Beyond informational benefits, the use of mobile exercise health applications may contribute to higher levels of exercise among cancer patients, with specific features (e.g. websites, gamification) potentially enhancing their overall effectiveness (30, 31). Thus, there is a need for interventional implementation research to evaluate the efficacy of these strategies in the context of increasing the number of cancer patients who participate in OET.

## Limitations

PA guidelines in oncology suggest 150 minutes/week of moderate-intensity activity (32). Despite the well-documented benefits of PA, most people undergoing cancer-therapy do not meet these PA guidelines (33). Yet in our sample, both women and men exceeded these recommendations, suggesting social desirability bias or recall inaccuracies. Additionally, the average weekly time spent on PA was higher than in a comparable German study (16). Most participants had a higher educational background, fewer had a migration background, and a higher proportion were privately insured compared to the general German population. Together with the low response rate (13%), this may limit the generalizability of the findings. Because some patients had been diagnosed some time ago, participants may have inaccurately reported past behaviors, which could potentially influence the true associations due to recall bias. Additional limitations include possible measurement error from unvalidated scales, the cross-sectional design preventing causal inference, and potential confounding due to unadjusted center effects. While validated instruments were used wherever available, some constructs had to be assessed using study-specific items due to a lack of established measures. Omitting a neutral option in a four-point Likert scale may lead to an inflation of the “agree” responses. The low consent proportion introduces a risk of selection bias, particularly as recruited participants appeared to be unusually motivated, highly active, and better informed than typical oncology patients. These factors may partly explain the comparatively low number of individual barriers reported for participating in OET. Nevertheless, data has been collected from different regions all over Germany and include patients with a variety of sociodemographic and disease-related characteristics and the reported rate of recommendations for PA by healthcare providers were similar to those in a previous study (16). However, as this was an exploratory study and not designed to yield a statistically representative sample, findings should be interpreted accordingly. Future research should aim to recruit even more heterogeneous samples and further validate measurement instruments to enhance internal and external validity.

## Conclusions

Our findings indicate that most cancer patients are both willing and able to participate in OET. However, they are not routinely informed about OET. The implementation of individually tailored resource-efficient communication strategies may facilitate the successful and sustainable integration of OET in routine care. Future research should focus on interventional studies to evaluate the effectiveness of such strategies in improving the implementation of OET.

## Data Availability

The raw data supporting the conclusions of this article will be made available by the authors, without undue reservation.
